# Supplementary material for: The impact of sport engagement on life satisfaction, mental and psychological well-being among athletes
Source: PLoS One. 2026 Jan 2;21(1):e0340069. doi: 10.1371/journal.pone.0340069 (PMC12758687; doi:10.1371/journal.pone.0340069)
Supplement: Attachment — Submitted filename: Response to reviewer.docx [file pone.0340069.s004.docx]

***Response to reviewer***

*Dear Reviewer, thank you for your consideration.*

Corrections and responses are listed below:

1. **Corrections regarding the introduction**

It was said in the introduction that the explanations about the positive effects of physical activity on general health were long. The sections describing the effects of physical activity on general health have been condensed into a short and concise sentence. *“In addition, the World Health Organization (WHO) and many other scientific studies emphasize the benefits of physical activity on general health.”* (See 47, 48, 49). Therefore, the transition to the basic structure of the research has been accelerated.

**The original aspect of the research, its rationale, and expected contributions;** *“In this context, the importance of this study is increasing by the emphasis placed on paticipation in physical activity in scientific studies, as well as the clear demonstration in reports published by the WHO (5) that a significant portion of health problems can be eliminated through paticipation in physical activity. This study, which examines life satisfaction and mental and psychological well-being levels, paticularly among active athletes, highlights the benefits of physical activity for individuals and society. It is anticipated that this study is essential for encouraging individuals to participate in physical avtivities. This study, which allows for a multifaceted assessment by considering the variables of sports engagement, sportive life satisfaction, mental and psychological well-being, which have been considered separately in previous studies, is considered original.”* (See 120, 121, 122, 123, 124, 125, 126, 127, 128, 129)

2. **Corrections to the method**

**2.1. Rationale for choosing the relational screening model;**

*“The reason for preferring the relational survey model is that, instead of looking for a cause-effect relationship between variables, the variables included in the model are more likely to have a common effect on individuals’ participation in physical activity and the changes that can be observed afterward.”* (See 140, 141, 142, 143).

**2.2. Consistency between hypotheses and the relational screening model;**

*“The hpyotheses of this study were tested with the variables and sample group selected in accordance with the relational survey model. It is thought that with the change in the sports engagement variable and its subdimensions, changes can also be seen in individuals’ sportive life satisfaction, mental and psychological well-being levels. Hypotheses were created from variables that have high potential to represent motivation to participate in physical activities and positive psychological changes that can be seen afterwards.”* (See 152, 153, 154, 155, 156, 157).

**2.3. Limitations of convenience sampling;**

*“On the other hand, there is a possibility of bias in the convenience sampling method because the sample selection can be determined by the researcher. Due to the lack of randomization, the convenience sampling method has questionable aspects in terms of generalizability.”* (See 172, 173, 174, 175).

**2.4. Effects of earthquake conditions;**

*“Although ethical approval for the study had previously been obtained, the suspension of sporting activities in the country due to the earthquake caused a delay in data collection. Because it was anticipated that the participating athletes would experience some social and psychological trauma due to the earthquake, data collection was delayed until sporting activities resumed and the athletes returned to routine physical activities. Given that the traumatic environment in which the athletes were located could be misleading, the data collection period was extended, and the study was conducted with athletes who were able to return to active sports.”* (See 176, 177, 178, 179, 180, 181, 182).

**2.5. Cultural adaptation methods of measurement tools;**

***2.5.1****.“****Sport Engagement Scale (SES):*** *Developed by Guillen & Martinez-Alvarado (17) and adapted to Turkish by Kayhan et al. (48), the SES measures sport engagement through two subdimensions: vigor and determination. This 10-item scale utilizes a 7-point Likert-type response format. During the adaptation process of the scale to Turkish, the opinions of 6 expert and correlation analysis were used and it was seen that the Turkish scale had language equivalence. To examine the construct validity of scale, EFA and CFA analyses were conducted with independent sample groups. KMO (0.94) and Barlett’ test of sphericity (𝜒2=3330,65, p<0,001) showed that the structure was suitable for discovery. It was observed that the sub-dimensions of the scale explained 67.59% of the total variance. Within the scope of CFA analysis, the fit indices of the scale (GFI=.95, TLI=.96, IFI=.97, RMSEA=.07 etc.) were examined and it was observed that they were within the acceptable fit range. The reliability of the scale was examined using Cronbach’ Alpha coefficients and Test-Re-Test (r=0.94) method. The Cronbach α coefficient of the whole scale was calculated as .91; Cronbach α coefficients of sub-scales are .91 and .77.”* (See 196, 197, 198, 199, 200, 201, 202, 203, 204).

***2.5.2.*** *“****Sportive Life Satisfaction Scale (SLSS):*** *Originally developed by Diener et al. (49) to assess general life satisfaction, this scale was adapted for the sports context by Mangan (50) and subsequently translated into Turkish by Somoglu & Cihan (26). The SLSS comprises 5 items, employs a 7-point Likert-type response style, and measures overall satisfaction with one's sporting life. In order to test the validity of the scale, content validity index (CVI), EFA and CFA analysis were examined. As a result of content validity analysis, the scale was found to represent the construct (CVI=0.96). Exploratory factor analysis showed that a one-factor structure explains 81.730% of the total variance. As a result of CFA (RMSEA=.057, SRMR=.011, GFI=.98, NFI=.99, RFI=.98, CFI=.99, IFI=.99), it was observed that the fit indices were within acceptable ranges. The values obtained from the results of Cronbach's Alpha (0.87) and Test-Re-Test (r=0.89) reliability analyzes showed that the scale is a reliable tool.”* (See 210, 211, 213, 214, 215).

***2.5.3.*** *“****Warwick-Edinburgh Mental Well-being Scale (WEMWBS):*** *The WEMWBS, developed by Tennant et al. (30) and adapted into Turkish by Keldal (51), is a 14 item scale assessing mental well-being through 5-point Likert responses. EFA and CFA analysis were conducted in independent sample groups to prove the validity of the scale. The scale was found to be suitable for factor analysis according to the results of KMO (.85) and Barlett’ test of sphericity (𝜒2=2110,179, p<0,001). As a result of the analysis, it was found that 51% of the total variance was explained and the scale was unidimensional as in the original form. With CFA analysis, fit indices (𝜒2/sd=3.71, NFI=.94, RFI=.93, IFI=.96, CFI=.96, NNFI=.95, RMR=.054) were examined and it was observed that they were within the acceptable fit range. Cronbach's Alpha internal consistency reliability coefficient of the scale was found to be 0.92.”* (See 219, 220, 221, 223, 224, 225).

***2.5.4.*** *“****Psychological Well-Being Scale (PWBS):*** *Developed by Diener et al. (52) and adapted into Turkish by Telef (53), the PWBS is an 8-item scale measuring psychological well-being on a single dimension. It employs a 7-point Likert-type response design. The scale was found to be suitable for factor analysis according to the results of KMO (.84) and Barlett’ test of sphericity (𝜒2=1007,633, p<0,01). As a result of the exploratory factor analysis, it was found that the total explained variance was 42% and that the items were grouped under one factor. With CFA analysis, fit indices (𝜒2/sd=4,645, NFI=.94, RFI=.92, IFI=.95, CFI=.95, RMR=.04, RMSEA=.08) were examined and it was observed that they were within the acceptable fit range. The reliability study indicated that the Cronbach alpha coefficient was .80.”* (See 228, 229, 231, 232, 233).

**2.6. Model assumptions and variables included in the regression;**

*“Prior to the main analyses, the data were first screened for missing values and outliers. Skewness and kurtosis values were examined to assess normality, with all values falling within the acceptable range of -1 to +1, which indicated that the assumption of normality was met (54, 55, 56). Confirmatory factor analysis and reliability analyses (Cronbach's Alpha (α)) were conducted on the research scales. Pearson correlation analysis was performed to examine the bivariate relationships between the study variables. Finally, multiple regression analysis was performed, controlling for relevant variables, to investigate the predicted relationships between commitment to sports, satisfaction with sporting life, and mental and psychological well-being. Finally, multiple regression analysis was performed, controlling for relevant variables, to investigate the predicted relationships between commitment to sports, satisfaction with sporting life, and mental and psychological well-being. The basic assumptions of regression analysis, such as normality, linearity, homogeneity, independence, multicollinearity and absence of outliers, were examined. The confidence interval was evaluated as 95%. In addition to descriptive statistics, hypotheses were tested with correlation and multiple regression analyses within the scope of the relational model.”* (See 238, 239, 240, 241, 242, 243, 244, 245, 246, 247, 248, 249).

**2.7. Analysis process flow chart;**

Research design

Ethical approval

Literature review

Data collection

Making data ready for analysis

Statistical analysis

Interpretation of findings

(See 158, 159).

3. **Corrections regarding the findings**

Tables 1 and 2 should be placed in the appropriate locations. The relevant tables have been placed in their desired locations. (See 184, 250).

Additionally, table descriptions have been corrected as it was requested that they be placed below the tables. (See 186, 251, 267, 281).

4. **Corrections to the discussion**

**4.1. The original contribution of the research;**

*“Existing literature highlights the effects of individuals participating in physical activity on general health indicators (2, 3, 4, 5, 11, 12). However, participation in physical activities and individuals' commitment to sports contain some differences. This study demonstrates that individuals who choose a physically active lifestyle are both physically and psychologically fit by placing physical activities at the center of their lives. Therefore, increasing social welfare will be possible with physically and mentally resilient individuals. The contribution of the research results to the literature is important in that the relationship observed in the current health indicators of individuals committed to sports opens the door to multidisciplinary inferences.”* (See 432, 433, 434, 435, 436, 437, 438, 439, 440).

**4.2. Reflecting the relational scanning model;**

*“In this context, when an increase in an individual's level of engagement with sports is observed, it can be assumed that there may also be an increase in their sportive life satisfaction. Indeed, there can be many sources of motivation for individuals to participate in physical activities. In fact, this motivation may be related to many other psychological indicators. Therefore, it is thought that individuals who participate in physical activity with vigorus and dedicated motivation are also more likely to achieve satisfaction in sports.”* (See 340, 341, 342, 343, 344, 345, 346).

*“Participation in physical activities may be related to the activation of individual self-control systems. Consistently following sports activities requires discipline. It is important to remember that the individual's mental health is also a significant factor in all these processes. Considering that mental states such as alertness and focus are important factors in sports engagement, it can be expected that individuals will also be sufficiently healthy mentally. Indeed, it is thought that individuals who show an increase in their level of engagement to sports may also experience an improvement in their mental health at the same time.”* (See 364, 365, 366, 367, 368, 369, 370, 371).

**4.3. Contextual interpretation of the findings;**

*“In various social and cultural structures, factors such as socio-economic level, state and government policies, geographical conditions, traditions, etc. can have an impact on individuals' participation in physical activities. Indeed, in Türkiye, where the research was conducted, there are cultural, social, and political factors that influence individuals' commitment to sports. In particular, the state's reward system for successful athletes and the support mechanisms that will enable successful athletes to work at various levels of sports (coaches, educators, managers, etc.) in the future are in a position to encourage commitment to sports. Sporting activities, which have been disrupted in recent years due to the pandemic and natural disasters, have quickly entered a recovery phase as a result of the policies implemented. In this context, administrative processes have been successfully implemented to ensure the sustainability of all sporting activities. Therefore, the authorities have not overlooked the importance of utilizing the positive effects of participation in physical activities on individuals as a means to eliminate the negative experiences.”* (See 400, 401, 402, 403, 404, 405, 406, 407, 408, 409, 410, 411, 412).

**4.4. Sample diversity, the impact of cultural factors on the results;**

*“Within the scope of the research, data were collected from athletes operating in various branches such as football, rugby, wrestling, kickboxing, archery, table tennis, taekwondo, volleyball, swimming, cycling, handball and karate. The diversity of sports disciplines is important in terms of reflecting the psychometric characteristics of different groups in the research. Likewise, the fact that the sample group is close to each other in terms of gender contributes to the research. It is thought that the fact that each sport branch has its own unique participant profile increases the possibility of generalizing the research results. The type of physical activity in which individuals with culturally distinct characteristics participate provides an opportunity to observe them psychologically. This study, which includes participants from diverse backgrounds such as different cultures, different sports disciplines, and different genders, is expected to provide insights across a wide range of perspectives based on its findings.”* (See 418, 419, 420, 421, 422, 423, 424, 425, 426, 427, 428, 429).

5. **Corrections regarding limitations**

*“On the other hand, the fact that participants were included in the study using a convenience sampling method may limit generalizability due to the lack of randomization. In addition, the earthquake disaster in Turkey caused trauma on society. Due to the earthquake, sporting activities have been suspended for a period of time and athletes' routine physical activities have been disrupted. Therefore, delays in the data collection process due to the athletes being expected to return to their routine sports activities and move away from the negative effects of the earthquake can be considered among the limitations of the research.”* (See 450, 451, 452, 453, 454, 455, 456).

6. **Corrections to recommendations**

*“It may be recommended that bureaucrats and sports administrators discuss the possibility of enabling individuals to pursue both academic and athletic careers simultaneously. Additionally, for a society composed of physically and mentally healthy individuals, integrating physical activities into all levels of the education system can be proposed to the relevant authorities. On the other hand, it may be advisable to support initiatives undertaken through a public-private partnership model in rural and socio-economically disadvantaged regions with limited access to sports facilities and practices.”* (See 470, 471, 472, 473, 474, 475).

*“During the training of coaches, educators and managers, it can be suggested that in addition to physical capacity development programs, it may be useful to implement training programs such as sports psychology, sports sociology, sports philosophy, sports pedagogy, etc.”* (See 477, 478, 479, 480).

*“. In this context, it may be recommended that relevant institutions organize parent orientation training at regular periods to raise awareness among families.”* (See 481, 482, 483).

*Sincerely…*

*Dr. Arif ÖZSARI*
